# Does the presence of arthroscopically detected stage 1–2 glenohumeral osteoarthritis have any clinical impact on the outcome of arthroscopic rotator cuff repairs?

**DOI:** 10.55730/1300-0144.5576

**Published:** 2022-10-10

**Authors:** Mehmet Ali TOKGÖZ, Tarık ELMA, Aliekber YAPAR, Mustafa ÖZER, Muhammet Baybars ATAOĞLU, Ulunay KANATLI

**Affiliations:** 1 Department of Orthopaedics and Traumatology, Faculty of Medicine, Gazi University, Ankara, Turkey; 2 Department of Orthopaedics and Traumatology, Private Medline Adana Hospital, Adana, Turkey; 3 Department of Orthopaedics and Traumatology, Antalya Training and Research Hospital, Antalya, Turkey; 4 Department of Orthopaedics and Traumatology, Meram Medical Faculty, Necmettin Erbakan University, Konya, Turkey; 5 Department of Orthopaedics and Traumatology, Faculty of Medicine, Gazi University, Ankara, Turkey; 6 Department of Orthopaedics and Traumatology, Faculty of Medicine, Gazi University, Ankara, Turkey

**Keywords:** Rotator cuff rupture, glenohumeral osteoarthritis, arthroscopic rotator cuff repair, functional outcomes

## Abstract

**Background/aim:**

Rotator cuff rupture (RCR) and glenohumeral osteoarthritis (GHO) are two common disorders of the shoulder joint. However, there are very few reports that examine the relationship between them. This study aimed to present at least two years’ clinical results of arthroscopic rotator cuff repair of full-thickness and massive tears accompanied by arthroscopically detected early-stage osteoarthritis.

**Materials and methods:**

From August 2016 to December 2017, three hundred and twenty patients with total or massive rotator cuff tears were evaluated retrospectively. Thirty-five patients who were determined as stage 1 and 2 according to the Outerbridge scale for cartilage lesions were found appropriate for investigation. Patients were assessed using the University of California Los Angeles (UCLA) score, and a visual analog scale (VAS) score before surgery and at the final follow-up. The American Shoulder and Elbow Surgeons (ASES) shoulder score was used to evaluate the final outcomes and compare the UCLA shoulder scores.

**Results:**

The UCLA scores increased from the preoperative value of 19.1 ± 3.2 to 29.8 ± 4.8 at the last follow-up and increased by an average of 10.7 ± 6.0 (p < 0.001). The median VAS score decreased from the preoperative value of 3.0 to 1.0 (p < 0.001). Besides, the mean ASES score was found as 80.2 ± 10.6. An excellent positive correlation was found between postoperative UCLA scores and ASES scores (r = 0.887; p < 0.001).

**Conclusion:**

To the best of our knowledge, this is one of the first arthroscopic comparative studies about the effect of early glenohumeral osteoarthritis on clinical outcomes after rotator cuff tear treatment. Finding good and excellent results up to 71% after RCR repair in patients with early-stage osteoarthritis was an indication that arthroscopic repair could be planned as the first-line treatment option for RCR pathologies in patients with early-stage degenerative arthritis without considering the rerupture rate.

## 1. Introduction

Rotator cuff rupture (RCR) and glenohumeral osteoarthritis (GHO) are shoulder disorders frequently encountered by orthopedic surgeons. However, there are few published reports on RCR repairs performed with the presence of GHO [1–3]. The frequency of both RCR and GHO increases with age. These diseases are expected to occur more frequently with the growing average life expectancy [4–6]. The frequency of the coexistence of GHO and RCR varies between 23% and 76% in the literature [3].

Previous studies provide controversial information about the relationship between these two common shoulder diseases [7]. The relationship between the severity of GHO and RCR has not yet been fully established [8]. Joint arthroplasty is a valuable treatment option in patients with severe RCR and GHO [9]; however, although many GHO cases accompanying RCR are not known to be severe, only a few studies have examined the effects of GHO on RCR treatment. In these studies, it was reported that GHO badly affected the clinical results and increased the frequency of rerupture [3,8]. In the literature, the arthroscopic repair is still recommended as a treatment option for full-thickness and massive RCR with early-stage osteoarthritis [10].

This study aimed to present at least two years’ clinical results of arthroscopic rotator cuff repair of full-thickness and massive tears accompanied by arthroscopically detected early-stage osteoarthritis.

## 2. Materials and methods

From August 2016 to December 2017, three hundred and twenty patients with total or massive rotator cuff tears that were treated with arthroscopic primary rotator cuff repair were evaluated retrospectively. Massive irreparable tears and partial rotator cuff tears were not included. Patients with fractures, extremity deformities, and previous upper limb surgeries or those having congenital, rheumatologic, endocrine, oncologic, or hematologic diseases were excluded from the study. The study was conducted in accordance with the principles of the Declaration of Helsinki, and the protocol was approved by the ethics committee from the institute of the current study (date: 14.07.2020; number: 07)

All patients who performed arthroscopy in our institute were investigated preoperatively by physical examination, x-rays, and MRI findings. Intra-articular findings such as GHO, SLAP, RCR, and lesion morphologies of the patients were confirmed arthroscopically and recorded. The rotator cuff repair database of our institute was retrospectively scanned by two senior orthopedic surgeons (U.K. and M.Ö) experienced in shoulder arthroscopy and clinical research.

Considering the entire case series of 320 patients, osteoarthritis was observed in 70 (22%) patients. When the Outerbridge scale for cartilage lesions [11] was examined, the distribution of the cases was as follows: Stage 1: 44 (62.9%), stage 2: 19 (27.1%), stage 3: 5 (7.1%), stage 4: 2 (2.9%). However, only patients with early-stage osteoarthritis who underwent repair were included in the current study. Patients with advanced osteoarthritis (4 patients) who underwent tendon repair, 21 patients with irreparable tendon tears and 10 patients with partial tears were not included in the study. Four patients were excluded from the study because they did not continue with postoperative follow-ups at proper times.

All surgical procedures were performed by the same surgeon under 5 kg of traction in the lateral decubitus position. The superior posterior part of the rotator cuff tear was repaired by using a double-row equivalent repair technique, and for subscapularis tears, we preferred to use a single-row repair technique. Acromioplasty was added to the procedure where subacromial impingement was observed in clinical and arthroscopic findings.

Patients were assessed using the University of California Los Angeles (UCLA) score, and a visual analog scale (VAS) score before surgery and at the final follow-up. The American Shoulder and Elbow Surgeons (ASES) shoulder score was used for the evaluation of final outcomes and comparison of the UCLA shoulder scores.

Statistical analysis was performed using the Statistical Package for the Social Sciences Ver. 22.0 (Chicago, IL) computer program. In statistical analyses, categorical variables are given as numbers and percentages, and continuous variables are presented with mean ± standard deviation (SD) and median (min-max value) for descriptive analyses. The Chi-square test was used for the comparison of categorical variables between groups. The conformity of continuous variables to normal distribution was evaluated using visual (histogram and probability graphs) and analytical methods (Kolmogorov-Smirnov/Shapiro-Wilk tests). The Mann-Whitney U test was used for the comparison of data sets whose variables were not normally distributed. The paired samples t-test was used for the comparison of preoperative and last follow-up data sets with normal distribution. The Wilcoxon signed-ranks test was used for the comparison of preoperative and last follow-up data sets that were not normally distributed. Pearson correlation analysis was used to test the association between age and scores. Correlation coefficients (rho) of r ≤ 0.30 mean there is a weak correlation; if r ranges from 0.30 to 0.50, it means there is a moderate correlation; and r ≥ 0.50 means that there is a strong correlation [12]. P < 0.05 was considered statistically significant.

## 3. Results

Considering the entire case series of 320 patients, osteoarthritis was observed in 70 (22%) patients. However, only patients with early-stage osteoarthritis who underwent repair were included in the current study. Patients with advanced osteoarthritis (4 patients) who underwent tendon repair, 21 patients with irreparable tendon tears, and 10 patients with partial tears were not included in the study.

A total of 31 patients, 9 (29%) males and 22 (71%) females, with an average age of 67.6 ± 7.3 (range, 48–80) years, were included in the study. The mean duration of post-op follow-up was 25.5 (range, 24–39) months. Table 1 presents the results of the demographic and clinical features of the patients.

After the reevaluation of the arthroscopy videos of the patients, SLAP lesions were present in 25.8% (n = 8) of the patients. Twelve patients were found to have accompanied spontaneous biceps rupture, and 19 patients (61.3%) underwent tenotomy. Eight patients had a full-thickness supraspinatus rupture, and 23 had massive repairable rotator cuff ruptures, including the supraspinatus, infraspinatus, and subscapularis. Nineteen patients out of twenty-two that had coracoacromial degeneration underwent acromioplasty. Twenty-three patients had subscapularis lesions, and sixteen (74.2%) of them were repaired.

The tendon-bone repair was performed in 13 patients, and tendon-tendon sutures were used for converting the rupture to the crescent type in 18 patients before fixing the tendon to the footprint with the suture anchors in a double-row equivalent tension band fixation technique. None of the patients underwent SLAP repairs, and tenotomy was preferred for those patients. The UCLA scores increased from the preoperative value of 19.1 ± 3.2 (range, 10–24) to 29.8 ± 4.8 (range, 13–35) at the last follow-up and increased by an average of 10.7 ± 6.0 (p < 0.001). The median VAS score decreased from the preoperative value of 3.0 (range, 1–10) to 1.0 (range, 0–4.0), and this difference was found significant (p < 0.001) (Table 2).

**Figure 1:**
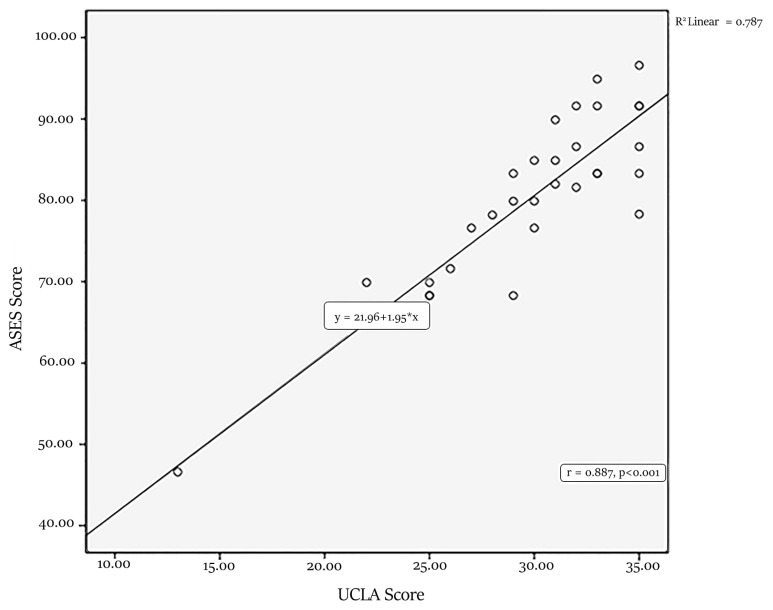
Correlation between last follow-up ASES and UCLA scores.

**Figure 2:**
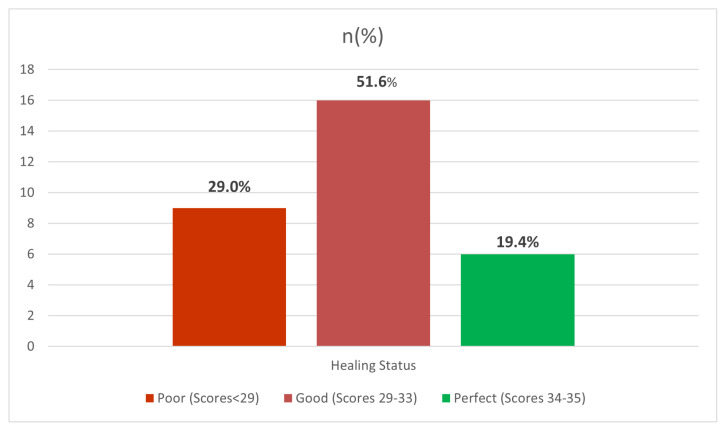
Distribution of healing status according to the last follow-up UCLA score.

It was determined that the UCLA shoulder score improved statistically significantly similar to the pain section VAS score (p: 0.011). Significant improvement was detected in the function section of the UCLA shoulder score (p: 0.001), shoulder active forward flexion (p: 0.008), and strength (p < 0.001). Besides, the ASES scores of the patients were evaluated at the last follow-up; the mean ASES score was found as 80.2 ± 10.6 (range, 46.6–96.6). An excellent positive correlation was found between postoperative UCLA scores and ASES scores (r = 0.887; p < 0.001) (Figure 1). When the functional and clinical improvements of the patients were evaluated according to the last follow-up UCLA scores, it was found that only nine patients (29%) had poor results, 16 patients (51.6%) showed promising results, and six patients (19.4%) showed excellent results (Figure 2).

It was found that the UCLA and ASES scores obtained at the last follow-up showed an excellent statistically significant positive correlation with the age of the patients (r = 0.440; p = 0.013 and r = 0.444; p = 0.007) (Table 3). According to this result, older patients were observed to have higher scores. Patients whose functional and clinical recovery status were evaluated according to the UCLA score were divided into two groups; those with poor recovery status (n = 9) and good/excellent status (n = 22). No relationship was found between the presence of massive tears and poor clinical outcomes. Patients with poor results had significantly lower last follow-up UCLA and ASES scores in the last follow-up, but their VAS scores were higher (p < 0.001, p < 0.001, and p = 0.001, respectively). Also, the changes in VAS and UCLA scores in the follow-ups were found to be significantly different between the groups. The change of the scores of the group with poor results was limited compared with the other group (p = 0.022 and p = 0.001).

## 4. Discussion

The results of this study showed that arthroscopic rotator cuff repair improved postoperative functional scores and reduced pain in patients with early-stage osteoarthritis. In previous studies, it was mostly reported that GHO was a negative prognostic factor for rotator cuff repair; however, osteoarthritis was evaluated radiologically, and patients were included without discrimination of severity [3]. To the best of our knowledge, the current study is the first to be evaluated arthroscopically.

In the study conducted by Kukkonen et al., 26.7% of patients who were accepted as stage 0 according to the Kellgren-Lawrence classification had arthroscopic degenerative cartilage changes [10]. The diagnostic performance of magnetic resonance imaging (MRI) in the detection of glenohumeral cartilage lesions has been reported in the literature only at a moderate level [13]. This positive difference found in the current study was thought to be due to the inclusion of patients with early-stage osteoarthritis and the fact that cartilage damage was evaluated arthroscopically rather than radiologically.

When rotator cuff repair was first described, it was presented as a procedure that should not be performed in patients aged over 65 years. However, today, RCR repair can be used as a treatment option for older patients since it has a minimally invasive arthroscopic technique, and the elderly have increasing health conditions [14,15]. In the current study, it was found that the improvement in functional results showed a positive correlation with age. This difference might be due to the insufficient number of patients under the age of 60 years (average age: 67.6 years) and the low functional expectation of the older group. However, the postoperative evaluation showed that both ASES and UCLA (all subsections and total points) scores gave good and excellent results in 71% of patients, and arthroscopic rotator cuff repair might be beneficial in this group of patients.

Massive RCRs are often thought to be fraught with structural failure and poor clinical outcomes [16,17]. However, the current study demonstrated that the results were rather satisfactory in the RCR group. This situation could be due to the high average age of the patient group and the lower functional expectations. Furthermore, good clinical results have been reported in patients undergoing tendon repair after massive RCR, as in the current study [18–20].

In this study, it was thought that the radiologic evaluation of GHO could be misleading because the authors evaluated GHO arthroscopically. However, the study included patients with early glenohumeral osteoarthritis who were at relatively low risk of progression. Variable results have been reported by studies on rotator cuff repair and the progression of glenohumeral osteoarthritis. Gerber et al. showed that 48% of patients progressed at least one degree after ten years of follow-up according to the Samilson and Prieto classification [21]. It was noted in another study that progression in glenohumeral arthritis remained minimal for degenerative cuff disease at the end of eight years, and this progress was not affected by the severity or enlargement of tears at intermediate points [7].

Although the data were collected prospectively, the retrospective design of the study and sample size, and the absence of a control group were the most important limitations of the current study. Also, the follow-up period was limited (an average of 25.5 months). The patients’ degree of osteoarthritis and range of motion degrees of the shoulder were not evaluated in the postoperative period either. Another limitation of the study was that we did not evaluate the healing rate of the repaired tendon and its complications. We mainly considered the clinical outcome of the treatment.

To the best of our knowledge, this is the first arthroscopic study of the effect of early glenohumeral osteoarthritis on clinical outcomes after rotator cuff tear treatment. Finding good and excellent results up to 71% after RCR repair in patients with early-stage osteoarthritis was an indication that arthroscopic repair could be planned as the first-line treatment option for RCR pathologies in patients with early-stage degenerative arthritis without considering the rerupture rate.

## Figures and Tables

**Table 1:** Baseline demographics and information about patients.

Characteristics	Total N = 31

**Age, year**	
Mean ± SD	67.6 ±7.3

**Sex, n (%)**	
Female	22 (71.0)
Male	9 (29.0)

**Follow-up time, month**	
Mean ± SD	25.5 ± 2.4

**Side, n (%)**	
Right	21 (67.7)
Left	10 (32.3)

**Repair type, n (%)**	
Tendon-Bone	13 (41.9)
Tendon-Tendon/Tendon Bone	18 (58.1)

**Outerbridge scale evaluation**	
Stage 1	21 (67.7)
Stage 2	10 (32.3)

**SLAP lesion, n (%)**	
No	23 (74.2)
Yes	8 (25.8)

**Pathology of biceps, n (%)**	
Spontaneous rupture	12 (38.7)
Tenotomy	19 (61.3)

**Rotator cuff rupture, n (%)**	
Full thickness	8 (25.8)
Massive	23 (74.2)

**Acromion degeneration, n (%)**	
No	9 (29.0)
Yes (acromioplasty was performed in 19)	22 (71.0)

**Pathology of subscapularis**	
No	8 (25.8)
Yes	23 (74.2)
SD: standard deviation	

**Table 2:** Comparison of the preoperative and postoperative clinical outcome.

N=31		Mean ± SD	Median	Mean difference	p
UCLA 1 (pain)	Preoperative	4.39 ± 1.8	4	−4.0	<0.001^1^
	Last follow-up	7.80 ± 1.8	8		
UCLA 2 (function)	Preoperative	5.81 ± 2.3	6	−3.0	<0.001^1^
	Last follow-up	8.06 ± 1.9	8		
UCLA 3 (sctive forward flexion)	Preoperative	3.48 ± 1.4	4	−1.5	0.002^1^
	Last follow-up	4.58 ± 0.5	5		
UCLA 4 (strength of forward flexion)	Preoperative	3.42 ± 0.9	3	−1.5	<0.001^1^
	Last follow-up	4.68 ± 0.5	5		
UCLA 5 (satisfaction)	Last follow-up	4.83 ± 0.9	5	–	-
UCLA Total*	Preoperative	17.1 ± 3.4	18.5	−8.5	<0.001^1^
	Last follow-up	25.1 ± 3.9	30.5		
VAS (during activity)	Preoperative	6.77 ± 1.7	8.0	5.0	<0.001^1^
	Last follow up	1.97 ± 1.1	2		
VAS (at rest)	Preoperative	3.9 ± 2.0	3	2.5	<0.001^1^
	Last follow-up	1.35 ± 0.9	1		

1 Wilcoxon signed-ranks test

* UCLA 5 was not taken into account while calculating the last follow-up UCLA Total score.

**Table 3:** Relationship between age and scores.

	Age	
N = 31	r	P^1^
**ASES score**	0.477	**0.007**
**UCLA score**	0.440	**0.013**

1 Pearson correlation analysis
